# Premorbid adjustment associates with cognitive and functional deficits in individuals at ultra-high risk of psychosis

**DOI:** 10.1038/s41537-022-00285-1

**Published:** 2022-10-07

**Authors:** Julie Lundsgaard, Tina Dam Kristensen, Christina Wenneberg, Maja Gregersen, Merete Nordentoft, Louise Birkedal Glenthøj

**Affiliations:** 1 Copenhagen Research Centre for Mental Health (CORE), Copenhagen University Hospital, Copenhagen, Denmark; 2 Centre for Neuropsychiatric Schizophrenia Research, CNSR, Mental Health Centre Glostrup, University of Copenhagen, Glostrup, Denmark; 3 Department of Psychology, University of Copenhagen, Copenhagen, Denmark

**Keywords:** Psychosis, Schizophrenia

## Abstract

Premorbid social and academic adjustment are important predictors of cognitive and functional performance in schizophrenia. Whether this relationship is also present in individuals at ultra-high risk (UHR) for psychosis is the focus of the present study. Using baseline data from a randomised clinical trial (*N* = 146) this study investigated associations between premorbid adjustment and neuro- and social cognition and functioning in UHR individuals aged 18–40 years. Patients were evaluated with the Premorbid Adjustment Scale (PAS) comprising a social and an academic domain. Using validated measures neurocognition was assessed in the domains of processing speed, executive function, attention, verbal learning and memory, visual learning and memory, and working memory along with estimated IQ. Social cognitive domains assessed were theory of mind, emotion recognition, and attributional bias. Functional assessment comprised the domains of social- and role functioning, functional capacity, and quality of life. Linear regression analyses revealed poor premorbid academic adjustment to be associated with poorer performance in processing speed, working memory, attention, full scale IQ, and verbal IQ. Poor premorbid social adjustment was associated with theory of mind deficits. Additionally, both premorbid adjustment domains were associated with social- and role functioning and quality of life. Corroborating evidence from schizophrenia samples, our findings indicate poor premorbid adjustment to correlate with deficits in specific cognitive and functional domains in UHR states. Early premorbid adjustment difficulties may therefore indicate a poor cognitive and functional trajectory associated with significant impairments in early and established psychotic disorders suggesting targets for primary intervention.

## Introduction

Poor premorbid adjustment is considered a key feature of psychotic disorders^1–7^ as it indicates the presence of neurodevelopmental anomalies prior to the onset of frank symptoms of psychosis^8^. More specifically, premorbid adjustment presents distinctive developmental trajectories, which, after onset of psychosis, follow paths of cognitive dysfunction and functional impairments^9^. Studies on psychotic disorders report associations between premorbid adjustment and functioning in the domains of global functioning, social functioning, and quality of life^10–13^. Moreover, cognitive dysfunction is one of the core features of schizophrenia spectrum disorders, and may be a likely marker of psychosis before the onset of the illness as demonstrated in familial high-risk (FHR) samples and general population samples^14,15^. Findings from schizophrenia studies reveal correlations between premorbid adjustment and general neurocognitive ability^3,16^, verbal learning and memory^8,17–19^, processing speed^8^, working memory^8,10,17^, executive function^8,20^, attention^20^, full scale IQ^3^, and verbal IQ^8^. Links have also been established between premorbid social adjustment and the social cognitive domains of social knowledge^21^, emotion recognition^21^, and theory of mind^22^. Notably, premorbid dysfunction is related to more severe post-onset cognitive impairments^16,22,23^. Premorbid adjustment difficulties may therefore indicate adverse clinical and cognitive trajectories in schizophrenia spectrum disorders and consequently represent an advantageous target for early intervention. However, while studies in clinical samples have shown associations between premorbid dysfunction and cognitive and functional impairments in individuals with schizophrenia^3,8,10,16–18,20,22,24^ these findings may be confounded by illness chronicity or antipsychotic use. Studying individuals before they reach a clinical psychotic state circumvents the confounds of living with a psychotic disorder. In this study, these individuals are considered at ultra-high risk (UHR) for psychosis thus constituting a clinical group with a subthreshold syndrome or a genetic vulnerability potentially leading to psychosis^25–27^. Poor premorbid adjustment has been established in UHR individuals^28^ and is a key feature of the premorbid period^29^. The importance of further elucidating on the premorbid phase in UHR state is accentuated by studies suggesting that poor premorbid academic and social adjustment may act as predictors of conversion to psychosis in UHR states^30–35^.

Similar to individuals with schizophrenia spectrum disorders, UHR individuals exhibit significant impairments in neuro- and social cognition^36–38^ as well as in functioning^39^. Additionally, neuro- and social cognitive dysfunction have consistently been linked to severe functional impairments in UHR individuals and individuals with established psychosis^40,41^; Hence, these processes may thus be distinct but closely connected processes. The cognitive and functional impairments in UHR individuals have been reported to be potential predictors of transition to psychosis^27,33,36,42–46^. Furthermore, while numerous longitudinal FHR studies and general population studies have demonstrated early impairments, including cognitive and functional impairments, in individuals who develop psychotic disorder and those at FHR of psychosis^5,15,47–53^ conversion rates are substantially lower compared to UHR samples^54–56^. The current UHR design, therefore, enables the study of associations between premorbid adjustment and neuro- and social cognition and functioning close to potential illness onset in a cohort more enriched for risk, hence potentially contributing with valuable information to future primary intervention strategies. The aim of this paper is thus to extend the current knowledge on the impact of poor premorbid adjustment in psychosis spectrum disorders by exploring the relationship between premorbid adjustment and multiple areas of cognition and functioning in UHR states.

On the basis of the abovementioned research in schizophrenia spectrum disorders that has established links between premorbid academic adjustment and neurocognition and premorbid social adjustment and social cognition, we conducted exploratory analyses hypothesising that 1) poor premorbid academic adjustment would relate to neurocognitive deficits, and 2) poor premorbid social adjustment would relate to social cognitive deficits in UHR states. Finally, we hypothesised poor premorbid adjustment in both academic and social domains would relate to deficits in all aspects of current functioning.

## Results

Of the 146 UHR participants in the study, 58.2% were females. They had a mean age of 23.92 (*SD* = 4.24). A majority (76%) fulfilled the Comprehensive Assessment of At-Risk Mental States (CAARMS)^26^ criteria of the attenuated psychotic symptom (APS), 20.5% fulfilled the criteria of APS + trait/state, 2.1% fulfilled the APS + brief limited intermittent psychotic symptom (BLIPS) criteria, and 1.4% fulfilled the trait/state criteria (See Table 1 for baseline characteristics).

**Table 1:** Clinical characteristics of ultra-high risk participants at baseline (*N* = 146).

Variable	Baseline *N* (%)
Female	85 (58.2)
CAARMS status	
- APS	111 (76.0)
- BLIPS	-
- Trait/state	2 (1.4)
- APS + trait/state	30 (20.5)
- APS + BLIPS	3 (2.1)
Ethnicity	
- High-income countries	140 (95.9)
- Low-income countries	6 (4.1)
	Mean (SD)
Age	23.92 (4.24)
Years of education	14.50 (2.74)
Estimated IQ (WAIS-III)	103.15 (12.29)

*APS* attenuated psychotic symptom, *BLIPS* brief limited intermittent psychotic symptom, *CAARMS* comprehensive assessment of at-risk mental states

### Associations between premorbid academic adjustment and cognition and functioning

The linear regression analyses revealed that lower scores on premorbid academic adjustment were associated with slower processing speed and poorer attention (*R*^*2*^ = 0.05 and 0.05). Furthermore, poor premorbid academic adjustment was associated with lower working memory, though with a very small effect (*R*^*2*^ < 0.01). By extension, this association did not remain significant after correcting for multiple comparisons. Premorbid academic adjustment did not predict verbal learning and memory, visual learning and memory, or executive functions. Moreover, premorbid academic adjustment was related to lower full-scale IQ and verbal IQ (*R*^*2*^ = 0.13 and 0.14, respectively), but not performance IQ. Lastly, premorbid academic adjustment was related to reduced social- and role functioning, and quality of life but not functional capacity (*R*^2^ in the range of 0.05–0.12; See Table 2).

**Table 2:** Linear regression analyses of the associations between the independent variables of premorbid adjustment and the dependent variables of neurocognition, social cognition, and functioning (*N* = 146) with age, gender, and medication (antipsychotic, antidepressant, mood stabilisers, or benzodiazepines) as covariates.

Predictors	Dependent variables	^a^*R*^2^	*B*	SE *B*	*β*	*t*	95% CI		*p*
							LL	UL	
PAS academic	Neurocognition								
	- Processing speed	**0.05**	**−15.85**	**5.82**	**−0.23**	**−2.73**	**−27.35**	**−4.35**	0.007*
	- Working memory	<**0.01**	**12.46**	**5.73**	**0.19**	**2.18**	**1.14**	**23.79**	**0.031**
	- Attention	**0.05**	**−0.07**	**0.03**	**0.23**	**−2.76**	**−0.13**	**−0.02**	0.007*
	- Verbal learning and memory	0.03	−1.55	4.38	−0.03	−0.35	−10.21	7.12	0.725
	- Visual learning and memory	−0.02	−3.28	3.99	−0.07	−0.82	−11.17	4.62	0.413
	- Executive functions	0.01	0.12	0.90	0.01	0.14	−1.66	1.90	0.892
	- IQ_fullscale	**0.13**	**−24.94**	**6.10**	**−0.32**	**−4.09**	**−37.00**	**−12.88**	≤0.001*
	- IQ_verbal	**0.14**	**−35.11**	**7.80**	**−0.36**	**−4.50**	**−50.54**	**−19.69**	≤0.001*
	- IQ_performance	<0.01	−12.32	**8.00**	−0.13	−1.54	−28.13	3.49	0.126
	Functioning								
	- GF-Social	**0.12**	**−2.08**	**0.51**	**−0.33**	**−4.09**	**−3.09**	**−1.07**	≤0.001*
	- GF-Role	**0.06**	**−1.92**	**0.60**	**−0.27**	**−3.22**	**−3.10**	**−0.74**	0.002*
	- AQoL-8D	**0.05**	**−0.24**	**0.08**	**−0.27**	**−3.26**	**−0.39**	**−0.10**	≤0.001*
	- HiSoC	0.05	−7.00	4.22	−0.17	−1.66	−15.37	1.38	0.100
PAS social	Social cognition								
	- Theory of mind	**0.04**	**−5.96**	**2.09**	**−0.24**	**−2.85**	**−10.10**	**−1.83**	0.005*
	- Latency of emotion recognition	0.15	367.54	281.20	0.10	1.31	−188.65	923.73	0.193
	- Accuracy of emotion recognition	−0.04	−1.69	3.55	−0.04	−0.48	−8.70	5.33	0.635
	- Attributional bias	<0.01	−1.04	0.95	−0.09	−1.09	−2.91	0.84	0.277
	Functioning								
	- GF-Social	**0.18**	**−2.33**	**0.44**	**−0.41**	**−5.32**	**−3.19**	**−1.46**	≤0.001*
	- GF-Role	**0.04**	**−1.40**	**0.54**	**−0.22**	**−2.59**	**−2.46**	**−0.33**	0.011*
	- AQoL-8D	**0.02**	**−0.16**	**0.07**	**−0.20**	**−2.34**	**−0.29**	**−0.03**	0.021*
	- HiSoC	0.04	−5.48	3.77	−0.15	−1.46	−12.96	2.00	0.149

Regression analyses significant at *p* ≤ 0.05 level before correction are given in bold.

*AQoL-8D* Assessment of Quality of Life, *CI* confidence interval, *GF-Role* Global Functioning Role scale;, *GF-Social* Global Functioning Social scale, *HiSoC* High Risk Social Challenge Task, *LL* lower level, *SE* standard error, *UP* upper level.

^a^Adjusted R^2^.

*Significant after correcting for multiple comparisons according to the Benjamini-Hochberg procedure^88^ using a false discovery rate (FDR) of 0.05.

### Associations between premorbid social adjustment and cognition and functioning

The linear regression analyses showed that lower scores on premorbid social adjustment were related to reduced theory of mind (*R*^2^ = 0.04) but not emotion recognition latency or accuracy, or attributional bias. Premorbid social adjustment was also associated with reduced social- and role functioning and poorer quality of life (*R*^2^ in the range of 0.02–0.18), but it did not relate to functional capacity (See Table 2).

## Discussion

Supporting our first hypothesis, we found that poor premorbid academic adjustment in UHR individuals was associated with deficits in the neurocognitive domains of processing speed, working memory, and attention, along with full-scale IQ and verbal IQ. However, when corrected for multiple comparisons, the association between premorbid academic adjustment and working memory was not significant. Nonetheless, the links between poor academic adjustment and the neurocognitive domains are compatible with studies in schizophrenia reporting associations between premorbid adjustment and the same domains^3,8,10,19,20^. These studies, though, also reported associations between premorbid adjustment and verbal learning and memory and executive function^3,8,10,19,20^ beyond our findings. The correlation between poor premorbid academic adjustment and worse processing speed and attention is in line with a growing number of studies relating processing speed and attention to poor functional trajectory in the UHR population^41,57–59^. This indicates that processing speed and attention may be key cognitive aspects associated with functional impairments in UHR. Supporting the potential central role of poor processing speed in psychosis spectrum disorders, processing speed has been speculated to underlie generalised cognitive impairments and thus represent an important target for intervention^60,61^. The finding of premorbid academic functioning relating to estimated full-scale IQ and verbal IQ corresponds to findings in schizophrenia samples^3,8^. Poor premorbid academic adjustment reflects lower performance in school and an aversion to attend school^62^. In that respect, our results on associations between premorbid academic adjustment and neurocognition may imply that poor premorbid adjustment may set in motion a series of events that result in impaired neurocognitive processes in more pronounced psychopathological stages, i.e., UHR and established psychosis. This hypothesis is supported by the maturational nature of cognitive processes such as processing skills, working memory, attention, and aspects of IQ^20,63–65^. However, the reverse causality could also be a valid perspective as neurodevelopmental disturbances may interfere with premorbid adjustment causing individuals not to thrive in school^10,16^. Regardless of the causality, our findings add to the notion that premorbid academic dysfunction across childhood and adolescence relates to neurocognitive dysfunction in putative prodromal and established psychotic disorders. This supports the idea of early manifestations of poor academic adjustment potentially being involved in early abnormal cognitive developmental trajectory specific to developing psychosis several years prior to illness onset^66^.

We did not find associations between poor premorbid academic adjustment and verbal learning and memory, executive functions, visual learning and memory, or performance IQ. This may be explained by the notion of early versus late neurodevelopmental processes in which some cognitive areas like processing speed^67^, working memory^68,69^, and attention^70^ are regarded as basic cognitive domains with early maturational attainment. Other higher-order abilities like executive functions and verbal skills are instead still being refined throughout young adulthood and hence might not be influenced by poor academic adjustment in early years^70,71^.

Partly corroborating our second hypothesis, we found poor premorbid social adjustment to be associated with social cognition albeit only in the domain of theory of mind, and not emotion recognition or attributional bias. The relationship between premorbid social adjustment and theory of mind is compatible with findings from a schizophrenia study showing a significant link between these domains^22^. Speculating, children or adolescents struggling with social functioning in their premorbid years might persist having theory of mind deficits at UHR stages since poor premorbid social adjustment involves having few friends (if any) or not enjoying spending time with peers. This potentially limits development of theory of mind entailing impaired abilities later in life. Unfortunately, we were not able to show a significant association between premorbid social adjustment and emotion recognition, which have been reported in a schizophrenia study^21^. Neither did we find an association between premorbid social adjustment and deficits in attributional bias, albeit this could be due to dissatisfying psychometric properties of available measures of this domain^72^. Moreover, it must be noted that we only assessed social cognitive function in three of the hypothesised four social cognitive domains^72^. Future studies should therefore examine the relationship between the premorbid adjustment scale and all four core aspects of social cognition to reach more firm conclusions on this potential relationship.

Lastly, in partial correspondence with our third hypothesis, we found both poor premorbid academic and social adjustment to relate to impairments in social- and role functioning and quality of life but not with respect to functional capacity. Hence, individuals exhibiting poor adjustment in school and/or with peers in their childhood and adolescence, may be at risk for a poor functional trajectory. This notion is supported by studies in the schizophrenia population reporting associations between premorbid adjustment and global functioning, social functioning, and quality of life, thus suggesting poor premorbid adjustment will persist at post-illness onset^10–13^. Contrary to the strong association between premorbid adjustment and social- and role functioning and quality of life, we only found a trending association with social skills functional capacity. This could be explained by the High Risk Social Challenge Task (HiSoC)^73^ being suboptimal due to issues with tolerability and acceptability^57^ warranting further studies on this association using other functional capacity measures.

The current study must be considered within the context of potential limitations. First, it should be acknowledged that use of the term “premorbid” in this paper and in the wider literature is debatable. It is generally used to refer to the period before onset of psychotic symptoms that clearly indicate presence of a diagnosable psychotic illness. It can be argued that aspects of social and academic adjustment before diagnosis simply reveal early signs of the disorders, hence not being genuinely premorbid. From this outlook, aspects of premorbid social adjustment could reflect early negative symptoms often occurring before onset of positive symptoms. Premorbid academic adjustment could likewise, at least partially, reflect early neurocognitive impairment perhaps being an essential part of psychosis. Potentially limiting the findings is the retrospective design of the PAS^62^, although predictive and concurrent validity of the instrument have been established^74^. However, applying the PAS in a UHR sample is advantageous as the individuals are close in age to the developmental periods of interest and, as beforementioned, they have not reached a manifested psychotic state thus circumventing the confounds of living with a psychotic disorder. These aspects may limit the potential recall bias on the PAS. We acknowledge that our sample may be an older age range compared to other UHR studies (e.g.^34^), which may impact generalisability of our findings to the entire UHR population. Additionally, it must be acknowledged that PAS_academic and PAS_social only explain a modest proportion of the variability on neurocognition and social cognition, respectively, as well as on functioning. Finally, including multiple outcome measures in our analyses may involve a potential risk for multiplicity involving the risk of significant associations being spurious. Hence, our findings should be evaluated within a hypothesis-generating, exploratory perspective with p-values close to 0.05 being appraised cautiously. However, as cognition and functioning are multifaceted constructs, it is relevant to include multiple measures capturing the different aspects of these domains.

To conclude, our findings indicate that premorbid adjustment relates to distinct neurocognitive, social cognitive, and functional aspects in the UHR population. Our findings thus highlight the relevance of focusing on premorbid adjustment in clinical research as well as in early intervention preventive approaches. In keeping with the abovementioned evidence from other studies, our findings point to the premorbid period in childhood and adolescence as being a time where neurodevelopmental vulnerability is evident. At this early stage the individuals could be particularly responsive to psychosocial or pharmacological intervention^48,75,76^. While the current study extends findings from schizophrenia samples to UHR samples on important links between poor premorbid adjustment and cognitive and functional deficits, future studies should replicate our findings. Future studies should also elucidate on poor premorbid adjustment in relation to multiple aspects of social cognition using psychometrically well-validated instruments.

## Methods

Data applied in this study were baseline data from a randomised clinical trial (RCT) examining the effect of cognitive remediation in UHR individuals. These analyses are thus secondary to the RCT. Recruitments were from psychiatric in- and outpatient facilities in the greater catchment area of Copenhagen, Denmark, from April 2014 to December 2017. Trial protocol was approved by the Committee on Health Research Ethics of the Capital Region Denmark (study: H-6-2013-015). All participants provided informed written consent prior to the study.

### Participants

The sample consisted of help-seeking individuals aged 18–40 years meeting one or more of the UHR criteria according to the CAARMS^26^: the APS group, the BLIPS group, and/or the trait and vulnerability group along with a significant drop in functioning or sustained low functioning for the past year. Individuals were excluded if they had a history of a psychotic episode of ≥1-week duration, experienced psychiatric symptoms explained by a physical illness with psychotropic effect (e.g., delirium) or acute intoxication (e.g., cannabis use), had a diagnosis of a serious developmental disorder such as Asperger’s syndrome or mental retardation with an IQ < 70, or currently were treated with methylphenidate.

### Assessments

#### Clinical

APS level was assessed with the CAARMS scale comprising a global rating scale (ranging 0–6 where 0 is no symptoms present) and a frequency scale (ranging 0–6 where 0 is no occurrence)^26^. A CAARMS composite score was calculated according to the formula (Iutc ∗ Futc) + (Inbi ∗ Fnbi) + (Ipa ∗ Fpa) + (Ids ∗ Fds)^77,78^ range 0–144. CAARMS composite score was computed by weighing intensity of symptoms by their frequency^79^.

#### Premorbid adjustment

Premorbid adjustment was evaluated using a Danish translation of the premorbid adjustment scale (PAS)^62^; An interview-based rating schedule for assessing functioning, particularly social and academic functioning, across four developmental stages: childhood (up to 11 years), early adolescence (12–15 years), late adolescence (16–18 years) and adulthood (19 years and counting). The social adjustment domain consists of items from three domains, a) sociability/withdrawal, b) peer relationships, and c) ability to form interpersonal and sexual ties (only assessed in adolescence). The academic adjustment domain consists of items from two domains: achievement in school and adaption to school. As this study did not focus on specific age periods, we used a PAS “composite” score assembling all four age periods into one overall premorbid stage. This means that we did not distinguish between childhood, early adolescence, late adolescence, and adulthood in the premorbid period. The general information section and adulthood subscale were not included in the statistical analysis to avoid including symptoms of the prodromal phase. Moreover, items of sociosexual adaption were not included since these do not cover childhood^19^. Based on participants’ own statements, each item is rated on a 7-point scale ranging from 0 to 6, with 0 indicating healthy function and 6 denoting maximum adjustment deficits. According to PAS the premorbid period was defined as ending six months before first psychiatric contact or before the appearance of psychosis-like symptoms. Predictive and concurrent validity of the PAS has shown to be satisfactory^74^.

#### Functioning

Four measures were included covering functional aspects of social- and role functioning, self-reported quality of life, and performance-based functional capacity social skills. The Global Functioning: Social and Role Scales^30^ were used to assess social- and role functioning with a range from 1 to 10. On these scales, higher scores indicate better functioning levels. The scales have been developed specifically to assess functioning in the putative prodromal state of psychosis with age-appropriate anchors for assessing functioning in UHR.

Quality of life was reported with the Assessment of Quality of Life (AQoL-8D)^80^, which is a self-report measure, including physical (e.g., independent living and pain) and psycho-social (e.g., happiness, relationships, and coping) overarching dimensions. A composite quality of life score was used in the analyses but domain scores can be extracted from the instrument. The AQoL-8D has been used in another large-scale UHR trial^81^. Finally, the performance-based test HiSoC^73^ was included, measuring functional capacity of social skills such as display of affect, odd behaviour and language, social interpersonal anxiety, and task engagement. It is a standardised videotaped task in which the participants are instructed to do a 45-second audition in a mock competition, with a grand money prize, on being the most interesting person in the country. The task’s 16 items are rated on a 5-point Likert scale with higher scores indicating better social skills. HiSoC has demonstrated excellent reliability and validity in UHR^82^.

#### Cognition

The neurocognitive tests included cover six core neurocognitive domains as stated in the National Institute of Mental Health Measurement and Treatment Research to Improve Cognition in Schizophrenia (MATRICS) battery^77^. List learning and symbol coding from the Brief Assessment of Cognition in Schizophrenia (battery indexes verbal learning and memory and processing speed. Tests from Cambridge Neuropsychological Test Automated Battery (CANTAB)^78^ were Paired Associate Learning (PAL) indexing visual learning and memory, Spatial Working Memory (SWM) indexing working memory, Stockings of Cambridge (SOC) indexing executive function, and Rapid Visual Information Processing indexing sustained attention. Except for PAL and SWM the measures are expressed in the same direction, meaning that higher scores indicate a better cognitive performance. PAL and SWM measure number of errors on the task; hence, on these tasks a lower score indicates a better performance. To cover the seventh domain of the MATRICS battery, social cognition, we included four social cognitive measures covering three social cognitive domains^72^: Theory of mind with The Awareness of Social Inference Test^83^; attributional bias with Social Cognition Screening Questionnaire^84^; and facial emotion recognition accuracy and latency with Emotion Recognition Task of the CANTAB battery^85^. Current IQ was estimated using four subtests from Danish Weschler Adult Intelligence Scale Version 3 (WAIS-III), namely Vocabulary, Similarities, Block Design, and Matrix reasoning^86^ which are known to correlate strongly with full-scale IQ^87^. Based on these four subscales three outcomes measured were: verbal intelligence, performance intelligence, and an overall measure combining these two subscales. WAIS-III was administered and scored according to the standardised procedures specified by its manual.

### Statistical analysis

Analyses were performed using SPSS version 25.0. Descriptive statistics were reported as means and standard deviations. Data were examined for normal distribution and outliers. Linear univariate regression analyses were performed to investigate associations between premorbid social and academic adjustment and the domains of neurocognition, social cognition, and functioning at baseline. Significance levels were set to *p* < 0.05. As we examined associations between premorbid adjustment and cognition and functioning, PAS_academic and PAS_social were set as independent variables in the regression analyses with covariates of age, sex, and medication (antipsychotic, antidepressant, mood stabilisers, or benzodiazepines). Measures of cognition and functioning were dependent variables. We included one independent variable with the covariates and one dependent variable per model. Significance was corrected for multiple comparisons using the Benjamin-Hochberg procedure^88^ with a false discovery rate of 0.05.

## Data Availability

The datasets generated and/or analysed during the current study are available from the corresponding author upon reasonable request.
